# The food environment as a cardiometabolic exposure: mechanisms, inequities, and interacting environmental risk

**DOI:** 10.3389/fnut.2026.1851911

**Published:** 2026-10-22

**Authors:** Pedro Rafael Vieira de Oliveira Salerno, Jiada Zhan, Ricardo J. Estrada-Mendizabal, Omar Aboukhatwa, Avery Hum, Mohamed Bassiony, Leonel Al Dakar, Gayatri Setia, Darcy Freedman, Sadeer Al-Kindi, Salil V. Deo, Weichuan Dong

**Affiliations:** 1 Department of Medicine, NYC Health + Hospitals/Elmhurst, Icahn School of Medicine at Mt. Sinai, Queens, NY, United States; 2 Nutrition and Health Sciences, Laney Graduate School, Emory University, Atlanta, GA, United States; 3 Department of Cardiology, NYC Health + Hospitals/Elmhurst, Icahn School of Medicine at Mt. Sinai, Queens, NY, United States; 4 Mary Ann Swetland Center for Environmental Health, Department of Population and Quantitative Health Sciences, Case Western Reserve University School of Medicine, Cleveland, OH, United States; 5 Center for Cardiovascular Computational and Precision Health (C3PH), Department of Cardiology, Houston Methodist DeBakey Heart & Vascular Center, Houston, TX, United States; 6 Center for Health & Nature, Houston, TX, United States; 7 Cardiovascular Research Institute, Case Western Reserve University School of Medicine, Cleveland, OH, United States; 8 School of Health and Wellbeing, University of Glasgow, Glasgow, United Kingdom; 9 Surgical Services, Louis Stokes Cleveland, Cleveland, OH, United States

**Keywords:** cardiovascular disease, environment, nutrition, risk factors, ultra processed food

## Abstract

**Background:**

The exposome framework has expanded understanding of how cumulative environmental exposures influence cardiometabolic health. Although air pollution, green space, and socioeconomic factors are increasingly recognized within this framework, the food environment remains less consistently conceptualized as a structural cardiometabolic exposure.

**Methods:**

This narrative review synthesizes current literature on food environment determinants, including availability, accessibility, affordability, and food insecurity, alongside common approaches used to operationalize food environments in research. We also review mechanistic and epidemiologic evidence linking unhealthy food environments, particularly those characterized by high exposure to ultra-processed foods, with cardiometabolic outcomes. Finally, we discuss interactions with walkability, air pollution, climate stressors, socioeconomic vulnerability, and emerging exposures such as microplastics.

**Results:**

Food environments shape dietary behaviors through structural constraints that influence food choice, purchasing, and consumption. Unhealthy food environments may promote higher intake of ultra-processed foods and lower intake of minimally processed foods, contributing to obesity, insulin resistance, type 2 diabetes, cardiovascular disease, and chronic kidney disease through interconnected metabolic, inflammatory, and vascular pathways. These effects frequently cluster with other adverse neighborhood conditions, amplifying persistent and cumulative cardiometabolic risk contributing to health inequities. Current research is limited by heterogeneous exposure definitions, cross-sectional designs, residual confounding, and underrepresentation of low- and middle-income settings.

**Conclusions:**

The food environment should be considered a major structural component of the cardiometabolic exposome. Improving its measurement and prioritizing longitudinal, equity-focused, and policy-relevant research will be essential to inform interventions that reduce cardiometabolic disease burden and disparities.

## 1 Introduction

There is increasing interest in analyzing the exposome as a framework to understand how cumulative environmental exposures shape cardiometabolic disease risk across the life course. While certain components of the exposome, such as air pollution, greenery, and socioeconomic factors have been extensively studied as contextual drivers of cardiovascular risk, the food environment remains comparatively fragmented in measurement and interpretation (1–3). Within this framework, the food environment represents a structurally embedded exposure that was defined by Downs et al. as the consumer interface with the food system, encompassing availability, affordability, convenience, promotion, nutritional quality, and sustainability of foods across wild, cultivated, and built spaces, all within broader sociopolitical and ecological systems (3). In this narrative review, intended as an educational synthesis, we aim to introduce key concepts about the food environment and frame it as a significant cardiometabolic exposure, while exploring its mechanisms, inequities, and interacting environmental risks. A conceptual overview of these relationships is presented in Figure 1. However, much of this evidence and conceptual framing originates in Western, high-income countries and may not transfer directly to low- and middle-income settings, many of which face a double burden of undernutrition and diet-related chronic disease (4, 5).

**Figure 1:**
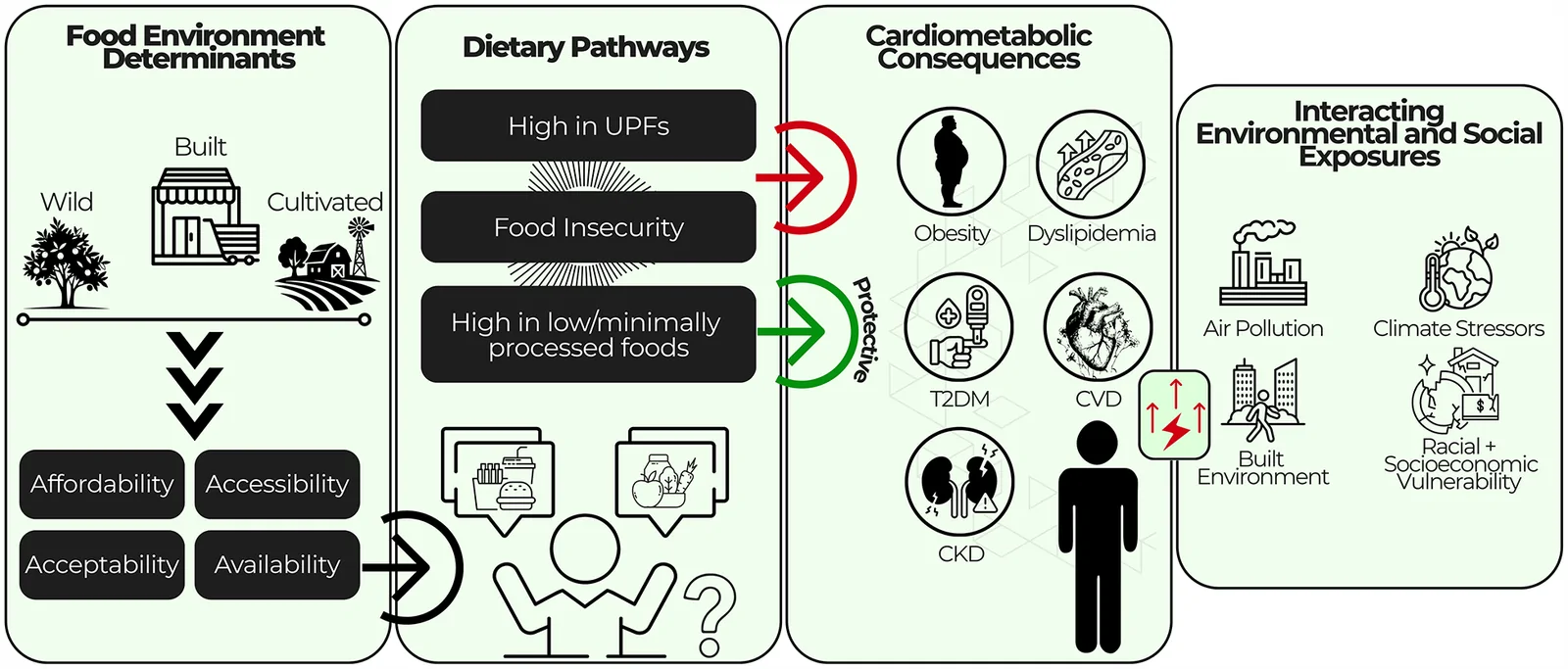
The food environment as a structural cardiometabolic exposure. Conceptual framework illustrating how structural characteristics of the food environment influence dietary behaviors and cardiometabolic health. Food environment determinants arise from multiple domains, including wild, cultivated, and built food systems, which together shape the availability, accessibility, and affordability of foods within a given context. These structural conditions influence dietary pathways by constraining or enabling food choices, contributing to patterns such as high consumption of ultra-processed foods (UPFs), food insecurity, or diets richer in minimally processed foods. These dietary patterns subsequently affect cardiometabolic risk through metabolic and inflammatory mechanisms, contributing to conditions including obesity, dyslipidemia, type 2 diabetes mellitus (T2DM), cardiovascular disease (CVD), and chronic kidney disease (CKD). The figure also highlights the role of broader environmental and social exposures that may amplify cardiometabolic risk. Factors such as air pollution, climate stressors, characteristics of the built environment, and racial or socioeconomic vulnerability frequently co-occur with adverse food environments, producing cumulative exposures that further exacerbate cardiometabolic disease risk and contribute to health inequities.

## 2 Food environment determinants

### 2.1 Availability, accessibility, affordability, acceptability, and food insecurity

Multiple dimensions of the food environment shape population dietary patterns and, by extension, cardiometabolic risk. It is important to recognize that an individual interacts with multiple food environment types, including wild and cultivated food environments, as well as built food environments that operate through both formal markets (licensed, regulated retailers such as supermarkets and franchised grocery chains) and informal markets (unlicensed or loosely regulated vendors) (3). This interaction is directly linked to closely related but conceptually distinct constructs: availability, accessibility, affordability, and acceptability (3, 6).

Availability refers to whether food vendors or products are present in a given context (3). In wild food environments, availability reflects local biodiversity and ecological conditions, with strong seasonal variation in what can be harvested (3). In cultivated environments, diversity depends on agricultural and climatic conditions, and on production practices such as crop selection, irrigation, mechanization, agrochemical use, and organic vs. conventional farming (3, 7, 8). In informal built environments, including wet markets (informal markets selling fresh, perishable foods) and small vendors (both fixed and mobile outlets such as street stalls, market kiosks, produce carts, and small independent shops), food availability may be extensive during harvest periods yet constrained by limited shelf life and limited diversity in smaller outlets (3). In formal built environments, such as the aptly named “supermarket” or franchised grocery stores availability may include a wide variety of foods across seasons sourced from multiple locations, but it also includes the broad penetration of minimally processed and ultra processed foods (UPF), with meaningful differences by neighborhood socioeconomic status in what is stocked and promoted (3, 5).

Accessibility is closely linked to availability, but it is a more individualized metric that extends from what is physically available to encompass what can actually be reached and used (3, 5). It is dynamic and includes distance and travel time, transportation options, physical mobility, safety, and the temporal structure of retail such as store hours (3, 5). Accessibility also represents the bridge between availability and affordability (3). The latter represents the intersection between food prices and household purchasing power (3).

Finally, acceptability captures whether foods that are available, accessible, and affordable are perceived as appropriate and worth choosing, meaning that the local supply aligns with people's personal standards. It is influenced by cultural preferences and taste, social norms, religious restrictions, preparation requirements, perceived quality and freshness, as well as perceived food safety (9, 10).

When food is not consistently available, accessible, affordable, or culturally acceptable, households may experience food insecurity, a condition tightly linked to diet quality, stress pathways, and downstream cardiometabolic risk (11). The U.S. Department of Agriculture (USDA) classifies food insecurity along a severity continuum, distinguishing low food security, characterized by reduced quality, variety, or desirability of diet with little or no indication of reduced food intake, from very low food security, characterized by disrupted eating patterns and reduced food intake (12).

Several geographic metaphors have been adopted to describe recurrent configurations of these structural constraints. Examples include food deserts, which describe areas where residents have limited access to affordable, nutritious food because outlets offering such food are sparse and distance, travel time, and transportation barriers constrain access (13). A food swamp, on the other hand, reflects areas in which there is a relative predominance of fast-food restaurants and convenience stores compared with healthier retailers such as supermarkets, greengrocers, and produce markets (14). These environments increase exposure to UPF and may promote convenience-driven consumption that shifts dietary defaults toward products that are inexpensive per calorie but metabolically unfavorable (15). Lastly, food mirages highlight the role of affordability within otherwise proximate food landscapes (13). In these settings, healthier outlets may be geographically available, yet prices exceed the purchasing power of low-income households (13). Proximity without price feasibility becomes functionally equivalent to constrained access, as households are compelled to seek lower-cost options that are often ultra-processed and nutrient-poor (13). Notably, these constructs are not mutually exclusive. A neighborhood may simultaneously exhibit limited spatial access, high density of unhealthy outlets, and price barriers within healthier stores. Additionally, these patterns of vulnerability do not arise randomly but are produced by structural forces, including residential segregation, oppression, economic disinvestment, and policy decisions that shape local food systems (16). Given the systemic and historically rooted nature of these inequities in food access and quality, this phenomenon has often been described as food apartheid (16).

Importantly, the operational definitions of these constructs, particularly food deserts and food swamps, vary widely across the literature and remain concentrated in high-income, largely Western settings, which limits comparison across studies and their transferability to other contexts (17).

### 2.2 Operationalizing the food environment in research

Operationalizing the food environment requires translating complex spatial, retail, and economic processes into measurable constructs. A variety of methodological approaches have been developed to characterize these environments, including spatial metrics, audit tools, retail environment indices, and large commercial and administrative datasets. These approaches capture complementary dimensions of the food environment, ranging from structural access to food retailers and in-store food availability to purchasing behaviors at population scales. Together, these methods provide a multidimensional framework for examining how food environments may influence dietary behaviors (18–21). Table 1 provides an overview of these approaches.

**Table 1:** Approaches for operationalizing the food environment.

Approach	Measures	Metrics	Tools/data sources
GIS-based spatial metrics	Structural access to food outlets	Outlet density and proximity to retailers	GIS datasets
Retail audit tools	In-store availability, pricing, and quality of foods	Healthy food availability scores, price comparisons, product placement	NEMS-S, NEMS-R, STORE
Retail food environment indices	Summary measures of healthy vs. unhealthy retail environments	Ratio of healthy to unhealthy retailers	RFEI, mRFEI
Diet quality linkage	Relationship between food environments and dietary quality	HEI scores linked to environmental exposures	HEI score
Administrative datasets	Regional food access and food security indicators	Supermarket access, food insecurity	USDA Food Environment Atlas
Commercial retail datasets	Food purchasing behavior and market trends	Product sales, market share, geographic sales patterns	NielsenIQ retail scanner data
Food processing classification	Nutritional quality and degree of industrial processing	Proportion of ultra-processed foods in diet or food supply	NOVA classification

GIS, geographic information system; NEMS-S, nutrition environment measures survey for stores; NEMS-R, nutrition environment measures survey for restaurants; STORE, stores observational tool for retail environments; RFEI, Retail Food Environment Index; mRFEI, Modified Retail Food Environment Index; HEI, Healthy Eating Index; USDA, United States Department of Agriculture.

#### 2.2.1 GIS-based spatial metrics

One of the most widely used approaches to operationalizing the food environment involves geographic information system (GIS)-based spatial metrics (22). These computational tools integrate spatial data with geographic coordinates to analyze patterns across locations (22). Spatial food environment metrics are commonly operationalized using measures of outlet density and proximity (22). Density refers to the number of food outlets (e.g., food stores or restaurants) within a defined geographic area. The most common method involves buffer analyses, which define a geographic zone around a location - such as a residence - to quantify the density or accessibility of nearby food retailers (22). On the other hand, proximity measures capture the distance between a reference location (e.g., home, school) and the nearest food outlet. This distance can be calculated using several distance metrics, including Euclidean (straight-line, 2 dimensions), Haversine (2-point distance accounting for the earth's curvature) Manhattan (city block), and network distance along road or transportation networks (22). In some studies, proximity is further operationalized as estimated travel time, which incorporates transportation modes, road characteristics, and geographic barriers to better capture the practical accessibility of food outlets (22). Spatial metrics may also incorporate store typologies to differentiate between food sources, such as supermarkets, grocery stores, convenience stores, fast-food restaurants, takeaways, and corner stores (19). These distinctions are important because different retail formats tend to offer distinct food profiles (23). Although GIS-based approaches provide valuable insights into structural access to food retailers, they primarily capture the potential availability of food sources rather than the nutritional quality or affordability of foods within those outlets (19, 22).

#### 2.2.2 Retail audit tools

To complement spatial metrics, several audit measures have been developed to assess the quality of foods available within retail outlets. Retail assessment tools provide a more detailed characterization of the consumer food environment by capturing within-store variability (20). One of the most widely used instruments is the nutrition environment measures survey for stores (NEMS-S), a standardized in-store audit tool designed to systematically evaluate the consumer food environment within retail food outlets (20). The NEMS-S assesses the availability, price, and quality of healthier food options across multiple product categories, including fruits, vegetables, dairy products, and whole grains (20). In addition to NEMS-S, several similar audit tools exist, such as the nutrition environment measures survey for restaurants (NEMS-R) and the stores observational tool for retail environments (STORE) (24, 25). By incorporating measures of pricing, product placement, and food quality, retail audits allow researchers to assess how the internal structure of food outlets influences purchasing behavior (20).

#### 2.2.3 Retail food environment indices

Retail food environment indices are summary metrics that characterize the overall retail food environment within a neighborhood. One example is the Modified Retail Food Environment Index (mRFEI), developed by the United States centers for disease control and prevention (CDC), which quantifies the relative availability of healthy food retailers within a geographic area (26). The mRFEI is calculated as the percentage of food retailers classified as healthy (e.g., supermarkets, large grocery stores, supercenters, and produce vendors) relative to the total number of food retailers in a given area, including less-healthy outlets (e.g., fast-food restaurants and convenience stores) (26). Such indices provide standardized metrics that facilitate comparisons across regions and population groups (26, 27).

#### 2.2.4 Commercial and administrative retail datasets

At broader geographic scales, researchers often rely on large administrative and commercial datasets to characterize food environments. In the United States, an example is the USDA Food Environment Atlas, which provides detailed county-level food-environment indicators, including supermarket access, food insecurity prevalence, participation in nutrition assistance programs, and food price measures (28). These datasets allow researchers to integrate food environment indicators with demographic, socioeconomic, and health outcome data (29). Additionally, commercial datasets are employed to characterize food purchasing patterns and product availability at large geographic scales. One example is the retail scanner data from NielsenIQ, which captures food sales across thousands of retail outlets, allowing researchers to analyze trends in food purchases, the market share of specific product categories, and geographic variation in food sales (30).

These datasets have important limitations. Commercial business listings are marketing products rather than research tools, and validation studies that compare them against ground-truthed field observations report substantial error: one field census found that Dun and Bradstreet undercounted 24% of existing supermarkets and grocery stores and InfoUSA 29%, with the undercount rising to 42 and 39% once errors in outlet-type classification were included (31). A meta-analysis of validation studies similarly found wide variability in accuracy and evidence that performance differs by population density and store type (32). Errors are not always evenly distributed, as public directory sources have been shown to characterize the food environment poorly in predominantly rural areas (33).

#### 2.2.5 Diet quality linkage

Dietary quality indicators, such as the Healthy Eating Index (HEI), are also used to examine the potential implications of food environments for dietary patterns. The HEI is a standardized scoring system developed by the USDA to evaluate how closely dietary patterns align with the Dietary Guidelines for Americans. Several versions of the index exist, including HEI-2015 and HEI-2020, which correspond to updates in the U.S. dietary guidelines (34). HEI scores are calculated by identifying the set of foods consumed by an individual or population, quantifying key dietary components (e.g., fruits, vegetables, whole grains, sodium, and added sugars), and assigning scores based on established intake standards (21, 34–36). The component scores are then summed to produce a total score ranging from 0 to 100, with higher scores indicating better adherence to dietary guidelines (21, 34–36). Another dietary index, American Cancer Society (ACS 2020) was created to assess diet quality related to highly processed food consumption and the quantity and diversity of fruits and vegetables and tested in a large racially, ethnically and geographically diverse US cohort (37).

Notably, there has been an increase in the development of open-source tools to facilitate standardized and reproducible implementation of these indices in research. For example, the R package dietaryindex provides user-friendly functions to compute multiple dietary indices across common datasets such as National Health and Nutrition Examination Survey (NHANES) (38).

#### 2.2.6 Food-processing classification

Another approach to characterizing dietary exposure within food environments involves classifying foods according to the degree of industrial processing. Food processing is a broad term encompassing various industrial, chemical, and biological processes, including grinding, milling, drying, cooking, frying, deboning, curing, fermenting, freezing, pasteurizing, sterilizing, and extrusion. These processes result in smaller particles and softer textures, improving palatability, digestibility, food safety, and shelf life (39, 40). The NOVA classification system categorizes foods by degree of processing into four groups: unprocessed or minimally processed foods (group 1), processed culinary ingredients (group 2), processed foods (group 3), and UPFs (group 4) (41). UPFs can undergo multiple industrial processes and commonly include cosmetic additives such as emulsifiers, flavor enhancers, sweeteners, and preservatives (40). Compared with minimally processed foods, UPFs tend to be energy-dense and nutrient-poor, containing high levels of refined sugars, sodium, and saturated or trans fats, alongside low fiber and micronutrient content (40, 42, 43). However, given that NOVA classifies foods according to the nature, extent, and purpose of processing rather than solely by nutrient composition. UPFs represent a heterogeneous category and should not be interpreted as having uniform nutritional profiles (44). In many high-income countries, UPFs account for approximately 42%−60% of total dietary intake (45, 46). As such, the NOVA classification has become an important framework for quantifying the contribution of UPFs within modern food environments and for examining their potential implications for dietary patterns and cardiometabolic health (40).

## 3 Biological mechanisms linking food environments to cardiometabolic risk

The mechanistic evidence discussed below primarily describes dietary and behavioral pathways through which food environments may influence cardiometabolic risk. Food environment characteristics represent upstream contextual exposures, whereas dietary intake, including UPF consumption and overall diet quality, represents a downstream behavioral pathway. Accordingly, evidence linking dietary exposures to cardiometabolic outcomes should not be interpreted as equivalent to direct causal evidence linking neighborhood food environments to clinical outcomes. The strength of evidence varies across pathways, with much of the food environment literature remaining observational and several proposed biological mechanisms supported primarily by experimental or preclinical studies.

The food environment is thought to shape dietary behaviors by influencing the types of foods people are exposed to, their accessibility and affordability, and the social and commercial contexts in which food choices are made (47, 48). Limited availability of fresh, minimally processed foods and greater exposure to UPFs may promote higher consumption of refined carbohydrates, added sugars, saturated fats, and sodium, while reducing intake of fiber, fruits, vegetables, and micronutrients (47–49). Poor geographic proximity to healthy food sources further encourages reliance on convenience food and meals consumed outside the home (37, 47). Elevated healthy food costs and poor individual purchasing power disproportionately steer individuals toward low-cost, calorie-dense foods with low nutritional value (11, 50, 51). These behaviors contribute to meal irregularity, larger portion sizes, and reduced dietary diversity (52).

Over time, consumption of UPF contributes to the development of metabolic syndrome, including excess adiposity, insulin resistance, dyslipidemia, and hypertension. Metabolic syndrome increases cardiovascular disease risk through multiple interrelated mechanisms (53). Insulin resistance impairs glucose uptake and promotes increased lipolysis in adipose tissue, leading to elevated circulating free fatty acids and accelerated atherogenesis (54, 55). In parallel, chronic hyperglycemia results in the formation of advanced glycation end products, which induce vascular injury and inflammation, further contributing to endothelial dysfunction, prothrombotic states, obesity, dyslipidemia, and hypertension (53, 54, 56). Sustained vascular injury ultimately manifests as organ-level pathology, including type 2 diabetes mellitus (T2DM), coronary artery disease (CAD), heart failure (HF), cerebrovascular disease, chronic kidney disease (CKD), and peripheral arterial disease (PAD) (53).

In addition, interactions between UPF consumption and the gut microbiome may amplify cardiometabolic risk via microbial metabolites such as trimethylamine N-oxide (TMAO) and delta-valerobetaine, which have been linked to CVD and obesity, respectively (57, 58).

### 3.1 Excess adiposity, insulin resistance, and diabetes

Unhealthy food environments may promote excess consumption of UPFs. From a mechanistic perspective, the composition of UPFs promotes adverse metabolic responses. Postprandial blood glucose levels are largely determined by the glycemic index (GI) of dietary carbohydrates, which reflects the speed at which carbohydrates are digested and absorbed. High-GI carbohydrates, prevalent in UPFs due to extensive refining and the addition of rapidly digestible sugars and starches, are quickly absorbed, leading to sharp increases in blood glucose and insulin levels (59, 60). Repeated postprandial insulin dysregulation promotes insulin resistance, pancreatic β-cell stress, and impaired glucose levels. In contrast, medium-GI carbohydrates elicit more stable glycemic responses, with metabolic effects that are determined by portion size and frequency of intake (61). Low-GI carbohydrates, typically found in minimally processed foods such as whole grains, legumes, vegetables, and fruits, retain structural integrity and fiber content, resulting in slower digestion and more gradual increases in blood glucose levels (43–45). Diets rich in low-GI carbohydrates are associated with improved insulin sensitivity and more stable glycemic control (62).

Multiple population-based studies across diverse settings consistently demonstrate a strong association between higher UPF consumption and increased risk of obesity and T2DM. In a large Canadian cross-sectional analysis using national nutrition survey data, individuals with the highest UPF intake (nearly three-quarters of total energy intake) had a 37% higher risk of diabetes compared with those with the lowest intake (63). These findings concur with prospective cohort studies. In a large French cohort followed for approximately 6 years, higher UPF consumption, assessed as a proportion of total food weight, was associated with incident T2DM, independent of overall dietary quality (64). Participants with greater UPF intake consumed more total energy, saturated fats, sodium, and sugars, alongside lower fiber intake. Similarly, a UK cohort study demonstrated a robust dose-response relationship, with each 10% increase in UPF consumption associated with a 12% higher risk of T2DM. Individuals in the highest UPF consumption group had a 44% greater risk of developing T2DM compared with those in the lowest group over a median follow-up of 5.4 years (43).

Beyond caloric surplus, UPF can disrupt gut microbiota composition, leading to reduced microbial diversity and inducing changes in specific bacterial populations. Additionally, UPFs can contribute to intestinal permeability, facilitating the translocation of bacterial lipopolysaccharides into the systemic circulation. This low-grade endotoxemia activates inflammatory pathways that impair insulin signaling in adipose tissue, liver, and skeletal muscle, thereby reinforcing insulin resistance and metabolic dysfunction (65, 66). Evidence suggests that food additives commonly found in UPFs, known as emulsifiers, may further exacerbate these processes. Emulsifiers can directly disrupt the gut mucus layer, alter microbial composition, and promote inflammatory responses independent of caloric intake. These effects have been associated with increased adiposity, glucose intolerance, and impaired metabolic homeostasis (65, 67).

### 3.2 Cardiovascular disease

Diet plays a key role in primary and secondary cardiovascular diseases (CVD) prevention, and is the leading factor related to the overall CVD burden in the US (68, 69). Epidemiologic studies have shown associations between higher consumption of UPFs and increased CVD risk (70–72). Accordingly, current American College of Cardiology and American Heart Association primary prevention guidelines recommend limiting UPF consumption (73).

The pathophysiological mechanisms linking UPF consumption to CVD involve multiple interconnected processes that are not fully understood. Evidence from experimental, mechanistic, and prospective epidemiologic studies supports a complex network of direct and indirect mechanisms (70). UPFs not only introduce nutritionally poor components, such as refined carbohydrates and added sugars, but also displace minimally processed, cardioprotective foods, including fruits and vegetables. Excessive dietary sodium intake is associated with an increased risk of hypertension, with UPFs representing a major source of added sodium in modern diets (72, 74). Elevated sodium intake promotes volume expansion, fluid retention, and increased arterial stiffness, contributing to higher blood pressure (74, 75). In contrast, higher dietary potassium intake has been associated with lower cardiovascular disease mortality, with favorable sodium-to-potassium ratios typically observed in minimally processed foods such as fruits, vegetables, and dairy products (70, 75).

The increased caloric load and content of high-GI carbohydrates associated with UPFs promote insulin resistance, which enhances adipose tissue lipolysis and hepatic production of atherogenic lipoproteins, contributing to elevated triglycerides and low-density lipoprotein cholesterol. Elevated low-density lipoprotein particles facilitate cholesterol transport into the arterial intima, where lipid accumulation leads to the formation of fatty streaks, the earliest morphological atherosclerotic lesions (55). Additional additives, such as emulsifiers, may also contribute to CVD risk (70). Furthermore, gut microbiome disruption and low-grade endotoxemia due to gut bacterial translocation can promote endothelial injury and contribute to a proinflammatory and prothrombotic state (65, 66).

These mechanistic pathways are supported by epidemiologic evidence linking UPF consumption to atherosclerotic cardiovascular disease and adverse cardiovascular outcomes. In a population-based prospective cohort study conducted in France, Sorur et al. followed 105,159 adult patients with dietary intake assessed using repeated 24-h dietary records, capturing usual consumption of more than 3,300 food items classified according to the NOVA processing framework (71). Over a median follow-up of 5.2 years, higher intake of UPFs was associated with increased incidence of overall CVD, CAD, and cerebrovascular disease (71). In the Tehran Lipid and Glucose Study, a prospective cohort of 2,050 Iranian adults aged ≥30 years without baseline cardiovascular disease, higher UPF intake was associated with increased cardiovascular disease incidence over 10.6 years of follow-up, with a 22% higher risk per 50 grams per day increase and a 68% greater risk among those in the highest intake tertile compared with the lowest (76). Moreover, an umbrella review including 14 meta-analyses and 9,888,373 total participants found a direct association between greater UPF consumption and higher risks of incident cardiovascular disease-related mortality (risk ratio 1.50, 95% confidence interval 1.37–1.63) (77).

### 3.3 Chronic kidney disease

CKD is a key component of the cardio-kidney-metabolic (CKM) syndrome and represents an important downstream manifestation of adverse food environments (78, 79).

Diets characterized by high consumption of UPFs contribute to CKD risk through metabolic, hemodynamic, and inflammatory pathways that overlap substantially with those driving CVD. Excess intake of refined carbohydrates and high-GI foods promotes insulin resistance and chronic hyperglycemia, leading to glomerular hyperfiltration, oxidative stress, and activation of profibrotic signaling within the renal microvasculature. These processes accelerate endothelial dysfunction and structural kidney injury over time (80–82). Moreover, preservatives used in UPF, such as sodium benzoate, potassium sorbate, and monosodium glutamate, have been shown to affect kidney function in animal models with significant increases in serum urea, creatinine, and uric acid (83).

Hypertension is a major determinant of CKD development and progression. Because UPFs are a major source of dietary sodium, they can contribute to renal hemodynamic stress through volume expansion and arterial stiffening, thereby increasing intraglomerular pressure and promoting progressive nephron loss (84). Moreover, alterations in the gut microbiome associated with UPF consumption may contribute to CKD pathophysiology. Emerging evidence suggests that diet-induced gut dysbiosis and low-grade endotoxemia may sustain systemic inflammation and promote endothelial injury across both renal and cardiovascular vascular beds. This interaction, often referred to as the “gut-kidney axis”, is thought to influence CKD progression through interconnected metabolic and immune pathways, although the current evidence remains limited (85).

Epidemiologic studies also indicate that UPF consumption is associated with the onset and progression of CKD. Prospective cohort analyses have shown that individuals in the highest quartiles of UPF intake have significantly higher risks of incident CKD compared with low consumers, independent of demographic, lifestyle, and clinical risk factors (86, 87). A meta-analysis of observational studies reports that high UPF intake is associated with a roughly 25% increased risk of incident CKD, and that each 10% increase in UPF intake corresponds to approximately a 7% greater risk of CKD (87). Additionally, evidence suggests that among individuals with preexisting CKD, greater UPF consumption is linked to faster disease progression and higher mortality (88).

## 4 Interactions with other social and environmental determinants

As mentioned, the cardiometabolic impact of the food environment does not occur in isolation. Rather, food access, affordability, and quality interact dynamically with other social and environmental determinants, shaping cumulative and synergistic exposures across the life course (3). Walkability, air pollution, climate stressors, socioeconomic vulnerability, and emerging exposures such as microplastics frequently co-occur with unhealthy food environments, reflecting shared structural drivers (1, 2, 89). These interactions amplify cardiometabolic risk beyond the effect of any single exposure and help explain persistent inequities in cardiometabolic disease burden across populations.

### 4.1 Walkability and food environment

Walkable neighborhoods which are characterized by connected street networks, mixed land use, and pedestrian-oriented infrastructure, facilitate both healthier food purchasing behaviors and greater levels of routine physical activity (89). In such settings, proximity to grocery stores, produce markets, and healthier retail options translates more effectively into improved dietary quality, as residents can access these resources without reliance on automobiles (90, 91). Conversely, car-dependent environments can, in part, undermine the benefits of healthy food availability as time spent in automobiles has been independently associated with higher obesity prevalence and adverse cardiometabolic profiles, while walkable environments promote habitual physical activity that supports energy balance (90). Even when nutritious foods are present, low walkability can restrict access for individuals without reliable and/or private transportation, particularly older adults, low-income populations, and those with disabilities (92). Evidence from built-environment studies demonstrates that walkability modifies the association between neighborhood food access and obesity, diabetes, and cardiovascular outcomes, suggesting a potential synergistic effect rather than an additive relationship (93).

Importantly, walkability is not evenly distributed across neighborhoods. Communities that have experienced long-term disinvestment, often due to historical zoning, transportation, and housing policies, are less likely to have sidewalks, safe street crossings, connected street networks, or nearby destinations that support walking (94). These same neighborhoods are also more likely to lack supermarkets and other sources of healthy foods, reflecting a shared policy and planning legacy rather than individual choice (94). When neighborhoods remain difficult or unsafe to navigate on foot, residents may still face barriers to accessing healthier food options and engaging in routine physical activity, as such, without parallel investments in pedestrian infrastructure and active transport, improvements in food retail environments may translate into smaller or inconsistent cardiometabolic benefits, particularly in low-income and historically marginalized communities (95).

### 4.2 Air pollution

Air pollution represents the leading environmental contributor to global morbidity and mortality, accounting for close to 10 million deaths each year worldwide, with cardiovascular disease responsible for the majority of this burden (1, 96, 97). Unhealthy food environments and air pollution frequently cluster within the same neighborhoods, reflecting shared structural determinants such as industrial zoning, highway placement, and historical disinvestment (98). Communities exposed to higher levels of particulate matter and traffic-related air pollution are also more likely to experience limited access to healthy foods and higher densities of fast-food outlets and convenience stores (98, 99). Additionally, some population studies suggest that diets rich in antioxidants and dietary fiber, often not ubiquitous, may blunt certain pollution-related cardiometabolic effects by reducing oxidative stress and systemic inflammation (100, 101). This way, the same structural inequities that concentrate air pollution exposure frequently coexist with poor food access, limiting dietary resilience and perpetuating cardiometabolic risk across generations.

### 4.3 Climate, heat, and food systems

Climate change introduces an additional layer of interaction between food environments and cardiometabolic risk. Rising temperatures and more frequent extreme heat events disrupt food systems through multiple pathways, including reduced agricultural productivity, interruptions in food distribution, degradation of food quality, and higher food prices (102). These effects disproportionately impact low-income communities, where food budgets are constrained and dietary substitutions often favor cheaper, energy-dense, UPF (102, 103).

Heat exposure also has direct adverse effects on cardiometabolic physiology (104). High ambient temperatures increase cardiovascular strain by raising heart rate, promoting dehydration, impairing thermoregulation, and triggering systemic inflammatory responses (105). When extreme heat occurs in neighborhoods with poor food environments, individuals face overlapping stressors including heightened physiologic vulnerability and reduced access to diets that could support metabolic resilience, especially in high risk geriatric populations (106, 107). Spatial analyses using composite indices demonstrate that heat exposure, food insecurity, and neighborhood deprivation frequently cluster geographically, identifying communities at particularly elevated risk for heat-related cardiovascular morbidity and mortality (106, 108).

Moreover, food retail environments themselves are sensitive to climate stress. Heat can reduce the shelf life of fresh produce, increase spoilage, and disrupt refrigeration, particularly in under-resourced stores (109). Extreme heat also constrains food access through household economic and behavioral pathways. Hot periods can reduce income among workers in heat-exposed and informal employment, lowering the capacity to purchase food, with the largest effects in lower-income settings (110). Heat can further disrupt food acquisition and preparation at the household level, shortening or deferring shopping trips, an effect that is most acute for households without a vehicle or cooling infrastructure and that shifts consumption away from perishable nutrient-rich foods toward shelf-stable products (111). Together, these pathways position climate change as a critical modifier of food environments and a key driver of widening cardiometabolic inequities.

### 4.4 Socioeconomic vulnerability

Socioeconomic conditions strongly influence how food environments affect cardiometabolic health. In communities facing poverty, the harmful effects of food apartheid are intensified by limited financial resources, which restrict food choices and increase reliance on inexpensive, calorie-dense foods (3, 112–114). At the same time, chronic constraints such as long working hours, job insecurity, and unstable housing reduce the time and resources available for food preparation and healthy eating, even when healthier food options exist nearby (112, 113, 115). Persistent poverty may also constrain access to transportation, which is particularly relevant in rural settings where longer travel distances are often required to obtain food (116).

Structural racism is a major driver of these patterns. Historical practices such as redlining, discriminatory housing and lending policies, and exclusionary zoning have shaped present-day neighborhood environments and cardiovascular burden (117, 118). Areas that were historically disinvested are more likely to lack supermarkets, have a higher concentration of fast-food outlets, and face greater exposure to environmental hazards such as air pollution and extreme heat (94, 117). The convergence of poor food access and adverse environmental and socioeconomic conditions creates a cumulative cardiometabolic burden that contributes to persistent health disparities across generations.

Socioeconomic vulnerability also affects how the body responds to diet and environmental stress. Chronic psychosocial stress linked to poverty and discrimination activates neuroendocrine and inflammatory pathways that promote visceral fat accumulation, insulin resistance, and elevated blood pressure (119).

### 4.5 Microplastics and packaging

Microplastics are increasingly recognized as a new environmental exposure that links modern food environments to cardiometabolic risk (120). UPFs are more likely than minimally processed foods to be packaged in plastic materials, which increases the potential for ingestion of micro- and nanoplastics through food contact, storage, and heating (121, 122). As consumption of UPFs increases, particularly in neighborhoods with limited access to fresh foods, dietary exposure to microplastics is likely to rise in parallel (121). Experimental and toxicological studies suggest that ingested microplastics may adversely affect metabolic health through several biological pathways, including oxidative stress, endocrine disruption, alterations in gut microbiota, and chronic low-grade inflammation (120, 123). These mechanisms overlap with established pathways involved in insulin resistance, obesity, and cardiovascular disease. Importantly, dietary intake appears to be a major route of microplastic exposure, directly linking food systems, packaging practices, and human health (120, 122).

## 5 Gaps and future directions

Most evidence linking food environments to cardiometabolic risk comes from North America, Western Europe, and other high-income settings, where food retail structures, pricing, and regulatory environments differ substantially from those in low- and middle-income countries (124, 125). This geographic concentration limits the generalizability of existing associations and limits the ability to compare findings across contexts using comparable metrics (124, 126). Expanding research to understudied regions is essential, but it will require locally valid and culturally sensitive measures that reflect dominant modes of food access, differences in packaged food penetration, and variability in food affordability (127). Without this broader representation, it remains difficult to determine whether observed relationships are universal features of cardiometabolic risk or context-dependent implications of specific food systems and policy environments (124, 126). This is particularly important as food environment research is especially vulnerable to confounding and measurement error because exposures are embedded within a broader web of social and environmental determinants which interact in complex ways (128, 129). Residential mobility, transportation access, safety, local economic conditions, and co-occurring exposures can obscure associations and, in some settings, may even invert the apparent direction of effects (128). Although many studies adjust for basic sociodemographic characteristics, approaches to confounding control are heterogeneous, and key sources of bias such as selective migration, differential access to healthcare, and unmeasured commercial determinants are often incompletely addressed (128, 129).

In addition, much of the available evidence remains cross-sectional, which limits temporal sequencing and strengthens the risk of ecological fallacy when group-level exposure metrics are used to infer individual behavior (126, 129). Static definitions of “neighborhood” based on administrative boundaries may not reflect actual exposure, particularly for individuals whose daily activity spaces extend beyond their residential area (129, 130). Small sample sizes, short follow-up, and inconsistent outcome definitions further complicate synthesis (126). Prospective designs with repeated exposure and outcome measurement are needed to capture the dynamic nature of food environments and to support more credible inference about long-term cardiometabolic effects (128, 129). Furthermore, there is limited longitudinal evidence examining the life-course and intergenerational effects of food insecurity and food environments, which may contribute to the persistence of cardiometabolic risk across generations (6).

Recent advances in geospatial methods, commercial data sources, and digital technologies create opportunities to improve food environment measurement at scale (126, 131). However, these tools also introduce challenges, including incomplete or inaccurate databases, limited information on food quality and affordability, and difficulties linking exposures to reliable, longitudinal cardiometabolic outcomes (126, 131). A collaborative framework is therefore essential. Multi-country research networks that share health outcomes data and measure food environments using common geospatial approaches could make findings easier to compare, enable pooled analyses, and speed the identification of which relationships are context-specific and which are broadly generalizable (124, 132).

At the same time, objective metrics alone may miss critical dimensions of exposure that shape behavior and risk, particularly among structurally marginalized groups (129). Proximity or density measures do not capture affordability, cultural acceptability, discrimination, safety, time scarcity, or the perceived accessibility of healthy options, all of which are factors that may determine whether a theoretically “healthy” retail environment translates into healthier diets (20, 129, 130). In addition, the rapid expansion of online and hybrid food purchasing environments introduces new challenges for exposure assessment, as these settings are dynamic, algorithm-driven, and not captured by traditional neighborhood-based measures (6). Future studies should integrate objective measures with community-informed and person-centered assessments, and utilize approaches that reflect daily mobility and purchasing patterns (20, 130, 131). This methodological blend can help clarify how both the material and experienced food environment influence dietary behaviors, stress-related pathways, and cardiometabolic outcomes.

Finally, translation of research requires stronger emphasis on policy-relevant designs. Natural experiments and quasi-experimental evaluations of retail activity, pricing policies, and nutrition assistance can provide more actionable evidence than cross-sectional associations alone (133–135). Aligning exposure metrics and outcome definitions across studies, embedding equity-focused analyses, and prioritizing scalable interventions will be key to building a more coherent evidence base and to inform policies capable of reducing cardiometabolic burden and disparities nationally and globally.

## 6 Conclusion

The food environment represents a structural determinant that influences cardiometabolic health through interconnected metabolic and inflammatory pathways. Importantly, these effects often interact with other environmental and social exposures such as walkability, air pollution, climate stressors, and socioeconomic vulnerability, producing cumulative cardiometabolic risk. Future research should prioritize improved measurement, longitudinal designs, and policy relevant evaluations to better understand how modifying food environments can reduce cardiometabolic disease and health inequities.

## References

[1] Rajagopalan S Brook RD Salerno PRVO Bourges-Sevenier B Landrigan P Nieuwenhuijsen MJ . Air pollution exposure and cardiometabolic risk. Lancet Diabetes Endocrinol. (2024) 12:196–208. doi: 10.1016/S2213-8587(23)00361-3 38310921 PMC11264310

[2] Motairek I Makhlouf MHE Rajagopalan S Al-Kindi S. The Exposome and Cardiovascular Health. Can J Cardiol. (2023) 39:1191–203. doi: 10.1016/j.cjca.2023.05.020 37290538 PMC10526979

[3] Downs SM Ahmed S Fanzo J Herforth A. Food environment typology: advancing an expanded definition, framework, and methodological approach for improved characterization of wild, cultivated, and built food environments toward sustainable diets. Foods. (2020) 9:532. doi: 10.3390/foods9040532 32331424 PMC7230632

[4] Popkin BM Corvalan C Grummer-Strawn LM. Dynamics of the double burden of malnutrition and the changing nutrition reality. Lancet Lond Engl. (2020) 395:65–74. doi: 10.1016/S0140-6736(19)32497-3 31852602 PMC7179702

[5] Turner C Aggarwal A Walls H Herforth A Drewnowski A Coates J . Concepts and critical perspectives for food environment research: a global framework with implications for action in low- and middle-income countries. Glob Food Secur. (2018) 18:93–101. doi: 10.1016/j.gfs.2018.08.003

[6] Odoms-Young A Brown AGM Agurs-Collins T Glanz K. Food insecurity, neighborhood food environment, and health disparities: state of the science, research gaps and opportunities. Am J Clin Nutr. (2024) 119:850–61. doi: 10.1016/j.ajcnut.2023.12.019 PMC10972712 38160801

[7] Kim DH Wade T Brym Z Ogisma L Bhattarai R Bai X . Assessing the agricultural, environmental, and economic effects of crop diversity management: a comprehensive review on crop rotation and cover crop practices. J Environ Manage. (2025) 387:125833. doi: 10.1016/j.jenvman.2025.125833 40412171

[8] Cozim-Melges F Ripoll-Bosch R. (Ciska) Veen GF Oggiano P Bianchi FJJA van der Putten WH . Farming practices to enhance biodiversity across biomes: a systematic review. Npj Biodivers. (2024) 3:1. doi: 10.1038/s44185-023-00034-2 39242701 PMC11332212

[9] Berkow SE Barnard N Eckart J Katcher H. Four therapeutic diets: adherence and acceptability. Can J Diet Pract Res. (2010) 71:199–204. doi: 10.3148/71.4.2010.199 21144137

[10] Farmer N Tuason R Middleton KR Ude A Tataw-Ayuketah G Flynn S . Assessing acceptability: the role of understanding participant, neighborhood, and community contextual factors in designing a community-tailored cooking intervention. Nutrients. (2024) 16:463. doi: 10.3390/nu16030463 38337747 PMC10857272

[11] Brandt EJ Mozaffarian D Leung CW Berkowitz SA Murthy VL. Diet and food and nutrition insecurity and cardiometabolic disease. Circ Res. (2023) 132:1692–706. doi: 10.1161/CIRCRESAHA.123.322065 37289902 PMC10882643

[12] Food Security in the U.S. - Definitions of Food Security. Economic Research Service. Available online at: https://www.ers.usda.gov/topics/food-nutrition-assistance/food-security-in-the-us/definitions-of-food-security (accessed March 1, 2026).

[13] Breyer B Voss-Andreae A. Food mirages: Geographic and economic barriers to healthful food access in Portland, Oregon. Health Place. (2013) 24:131–9. doi: 10.1016/j.healthplace.2013.07.008 24100236

[14] Lucan SC Maroko AR Patel AN Gjonbalaj I Elbel B Schechter CB. Healthful and less-healthful foods and drinks from storefront and non-storefront businesses: implications for ‘food deserts', ‘food swamps' and food-source disparities. Public Health Nutr. 23:1428–39. doi: 10.1017/S1368980019004427 32223780 PMC7196006

[15] Gearhardt AN Bueno NB DiFeliceantonio AG Roberto CA Jiménez-Murcia S Fernandez-Aranda F. Social, clinical, and policy implications of ultra-processed food addiction. The BMJ. (2023) 383:e075354. doi: 10.1136/bmj-2023-075354 37813420 PMC10561019

[16] Valle GR Guthey GT. From Food Deserts to Food Apartheid: Origins, Histories, and Solutions. Available online at: https://direct.mit.edu/books/oa-edited-volume/6088/chapter/5953423/From-Food-Deserts-to-Food-Apartheid-Origins (accessed March 31, 2026). doi: 10.7551/mitpress/15847.003.0012

[17] Ares G Turra S Bonilla L Costa M Verdier S Brunet G . WEIRD and non-consensual food deserts and swamps: a scoping review of operational definitions. Health Place. (2024) 89:103315. doi: 10.1016/j.healthplace.2024.103315 39013213

[18] Wilkins EL Morris MA Radley D Griffiths C. Using Geographic Information Systems to measure retail food environments: Discussion of methodological considerations and a proposed reporting checklist (Geo-FERN). Health Place. (2017) 44:110–7. doi: 10.1016/j.healthplace.2017.01.008 28236788

[19] Caspi CE Sorensen G Subramanian SV Kawachi I. The local food environment and diet: a systematic review. Health Place. (2012) 18:1172–87. doi: 10.1016/j.healthplace.2012.05.006 22717379 PMC3684395

[20] Glanz K Sallis JF Saelens BE Frank LD. Nutrition environment measures survey in stores (NEMS-S): development and evaluation. Am J Prev Med. (2007) 32:282–9. doi: 10.1016/j.amepre.2006.12.019 17383559

[21] Krebs-Smith SM Pannucci TE Subar AF Kirkpatrick SI Lerman JL Tooze JA . Update of the healthy eating index: HEI-2015. J Acad Nutr Diet. (2018) 118:1591–602. doi: 10.1016/j.jand.2018.05.021 30146071 PMC6719291

[22] Charreire H Casey R Salze P Simon C Chaix B Banos A . Measuring the food environment using geographical information systems: a methodological review. Public Health Nutr. (2010) 13:1773–85. doi: 10.1017/S1368980010000753 20409354

[23] Cooksey-Stowers K Schwartz MB Brownell KD. Food swamps predict obesity rates better than food deserts in the United States. Int J Environ Res Public Health. (2017) 14:1366. doi: 10.3390/ijerph14111366 29135909 PMC5708005

[24] Saelens BE Glanz K Sallis JF Frank LD. Nutrition environment measures study in restaurants (NEMS-R): Development and Evaluation. Am J Prev Med. (2007) 32:273–81. doi: 10.1016/j.amepre.2006.12.022 17383558

[25] Cohen DA Schoeff D Farley TA Bluthenthal R Scribner R Overton A. Reliability of a store observation tool in measuring availability of alcohol and selected foods. J Urban Health Bull N Y Acad Med. (2007) 84:807–13. doi: 10.1007/s11524-007-9219-x 17763963 PMC2232033

[26] Census Tract Level State Maps Of The Modified Food Environment Index (mRFEI). 2012. Available online at: https://stacks.cdc.gov (accessed March 5, 2026).

[27] Ahalya M Jane YP Éric R Marc L Tina M Leia MM. Geographic retail food environment measures for use in public health. Health Promot Chronic Dis Prev Can Res Policy Pract. (2017) 37:357–62. doi: 10.24095/hpcdp.37.10.06 PMC5674767 29043763

[28] Food Environment Atlas. Economic Research Service. Available online at: https://www.ers.usda.gov/data-products/food-environment-atlas (accessed March 5, 2026).

[29] Food Environment Atlas. Economic Research Service. Available online at: https://www.ers.usda.gov/data-products/food-environment-atlas (accessed March 5, 2026).

[30] NIQ. NIQ - The Full View^*TM*^ *of Consumer Intelligence*. Available online at: https://nielseniq.com/global/en/ (accessed March 5, 2026).

[31] Liese AD Barnes TL Lamichhane AP Hibbert JD Colabianchi N Lawson AB. Characterizing the food retail environment: impact of count, type, and geospatial error in 2 secondary data sources. J Nutr Educ Behav. (2013) 45:435–42. doi: 10.1016/j.jneb.2013.01.021 23582231 PMC3713101

[32] Lebel A Daepp MIG Block JP Walker R Lalonde B Kestens Y . Quantifying the foodscape: a systematic review and meta-analysis of the validity of commercially available business data. PLoS ONE. (2017) 12:e0174417. doi: 10.1371/journal.pone.0174417 28358819 PMC5373546

[33] Longacre MR Primack BA Owens PM Gibson L Beauregard S Mackenzie TA . Public directory data sources do not accurately characterize the food environment in two predominantly rural states. J Am Diet Assoc. (2011) 111:577–82. doi: 10.1016/j.jada.2011.01.008 21443992 PMC3119892

[34] Healthy Eating Index (HEI). Food and Nutrition Service. Available online at: https://www.fns.usda.gov/cnpp/healthy-eating-index (accessed March 5, 2026).

[35] NIH. Available online at: https://epi.grants.cancer.gov/hei/developing.html (accessed March 5, 2026).

[36] Shams-White MM Pannucci TE Lerman JL Herrick KA Zimmer M Meyers Mathieu K . Healthy eating index-2020: review and update process to reflect the dietary guidelines for Americans, 2020–2025. J Acad Nutr Diet. (2023) 123:1280–8. doi: 10.1016/j.jand.2023.05.015 37201748 PMC10524328

[37] McCullough ML Chantaprasopsuk S Islami F Rees-Punia E Um CY Wang Y . Association of socioeconomic and geographic factors with diet quality in US Adults. JAMA Netw Open. (2022) 5:e2216406. doi: 10.1001/jamanetworkopen.2022.16406 35679041 PMC9185183

[38] Zhan JJ Hodge RA Dunlop AL Lee MM Bui L Liang D . Dietaryindex: a user-friendly and versatile R package for standardizing dietary pattern analysis in epidemiological and clinical studies. bioRxiv. (2023) 2023.08.07.548466. doi: 10.1101/2023.08.07.548466 PMC11600030 39182618

[39] Ultra-Processing or Oral Processing? A Role for Energy Density and Eating Rate in Moderating Energy Intake from Processed Foods - ScienceDirect. Available online at: https://www.sciencedirect.com/science/article/pii/S2475299122119876?utm_source=chatgpt.com (accessed January 29, 2026) 10.1093/cdn/nzaa019 PMC7042610 32110771

[40] Almarshad MI Algonaiman R Alharbi HF Almujaydil MS Barakat H. Relationship between ultra-processed food consumption and risk of diabetes mellitus: a mini-review. Nutrients. (2022) 14:2366. doi: 10.3390/nu14122366 35745095 PMC9228591

[41] Petrus RR. do Amaral Sobral PJ, Tadini CC, Gonçalves CB. The NOVA classification system: a critical perspective in food science. Trends Food Sci Technol. (2021) 116:603–8. doi: 10.1016/j.tifs.2021.08.010

[42] Martínez Steele E Popkin BM Swinburn B Monteiro CA. The share of ultra-processed foods and the overall nutritional quality of diets in the US: evidence from a nationally representative cross-sectional study. Popul Health Metr. (2017) 15:6. doi: 10.1186/s12963-017-0119-3 28193285 PMC5307821

[43] Levy RB Rauber F Chang K Louzada ML da C Monteiro CA Millett C . Ultra-processed food consumption and type 2 diabetes incidence: a prospective cohort study. Clin Nutr. (2021) 40:3608–14. doi: 10.1016/j.clnu.2020.12.018 33388205

[44] Louie JCY. Are all ultra-processed foods bad? A critical review of the NOVA classification system. Proc Nutr Soc. (2025) 4:1–9. doi: 10.1017/S0029665125100645 40757421

[45] Rauber F da Costa Louzada ML Steele EM Millett C Monteiro CA Levy RB. Ultra-processed food consumption and chronic non-communicable diseases-related dietary nutrient profile in the UK (2008^−^2014). Nutrients. (2018) 10:587. doi: 10.3390/nu10050587 29747447 PMC5986467

[46] Machado PP Steele EM Levy RB Sui Z Rangan A Woods J . Ultra-processed foods and recommended intake levels of nutrients linked to non-communicable diseases in Australia: evidence from a nationally representative cross-sectional study. BMJ Open. (2019) 9:e029544. doi: 10.1136/bmjopen-2019-029544 31462476 PMC6720475

[47] Council (US) NR. Summary. In: The Public Health Effects of Food Deserts: Workshop Summary. National Academies Press (US); 2009. Available online at: https://www.ncbi.nlm.nih.gov/books/NBK208018/ (accessed January 29, 2026). 25032337

[48] Serafim P Borges CA Cabral-Miranda W Jaime PC. Ultra-Processed Food Availability and Sociodemographic Associated Factors in a Brazilian Municipality. Front Nutr. (2022) 9:858089. doi: 10.3389/fnut.2022.858089 35529462 PMC9067397

[49] Ultraprocessed Foods Account for More than Half of Calories Consumed at Home. Johns Hopkins. Bloomberg School of Public Health. 2024. Available online at: https://publichealth.jhu.edu/2024/ultraprocessed-foods-account-for-more-than-half-of-calories-consumed-at-home (accessed January 29, 2024).

[50] Leung CW Fulay AP Parnarouskis L Martinez-Steele E Gearhardt AN Wolfson JA. Food insecurity and ultra-processed food consumption: the modifying role of participation in the supplemental nutrition assistance program (SNAP). Am J Clin Nutr. (2022) 116:197–205. doi: 10.1093/ajcn/nqac049 35199832 PMC9257471

[51] Aljahdali AA. Food insecurity and ultra-processed food consumption in the health and retirement study: cross-sectional analysis. J Nutr Health Aging. (2025) 29:100422. doi: 10.1016/j.jnha.2024.100422 39778268 PMC12179980

[52] Mozaffarian D Aspry KE Garfield K Kris-Etherton P Seligman H Velarde GP . “Food is medicine” strategies for nutrition security and cardiometabolic health equity: JACC state-of-the-art review. J Am Coll Cardiol. (2024) 83:843–64. doi: 10.1016/j.jacc.2023.12.023 38383100

[53] Hamooya BM Siame L Muchaili L Masenga SK Kirabo A. Metabolic syndrome: epidemiology, mechanisms, and current therapeutic approaches. Front Nutr. (2025) 12:1661603. doi: 10.3389/fnut.2025.1661603 40969606 PMC12441046

[54] Razani B Chakravarthy MV Semenkovich CF. Insulin resistance and atherosclerosis. Endocrinol Metab Clin North Am. (2008) 37:603–21. doi: 10.1016/j.ecl.2008.05.001 18775354 PMC2639785

[55] Mundi S Massaro M Scoditti E Carluccio MA van Hinsbergh VWM Iruela-Arispe ML . Endothelial permeability, LDL deposition, and cardiovascular risk factors—a review. Cardiovasc Res. (2017) 114:35–52. doi: 10.1093/cvr/cvx226 29228169 PMC7729208

[56] Ormazabal V Nair S Elfeky O Aguayo C Salomon C Zuñiga FA. Association between insulin resistance and the development of cardiovascular disease. Cardiovasc Diabetol. (2018) 17:122. doi: 10.1186/s12933-018-0762-4 30170598 PMC6119242

[57] Liu KH Owens JA Saeedi B Cohen CE Bellissimo MP Naudin C . Microbial metabolite delta-valerobetaine is a diet-dependent obesogen. Nat Metab. (2021) 3:1694–705. doi: 10.1038/s42255-021-00502-8 34931082 PMC8711632

[58] Wang Z Klipfell E Bennett BJ Koeth R Levison BS DuGar B . Gut flora metabolism of phosphatidylcholine promotes cardiovascular disease. Nature. (2011) 472:57–63. doi: 10.1038/nature09922 21475195 PMC3086762

[59] Peppa M Manta A Mavroeidi I Nastos C Pikoulis E Syrigos K . Dietary approach of patients with hormone-related cancer based on the glycemic index and glycemic load estimates. Nutrients. (2023) 15:3810. doi: 10.3390/nu15173810 37686842 PMC10490329

[60] Mariamenatu AH Abdu EM. Overconsumption of omega-6 polyunsaturated fatty acids (PUFAs) versus deficiency of omega-3 PUFAs in modern-day diets: the disturbing factor for their “balanced antagonistic metabolic functions” in the human body. J Lipids. (2021) 2021:8848161. doi: 10.1155/2021/8848161 33815845 PMC7990530

[61] Sanpawithayakul K Kaewprasert N Tantiyavarong P Wichansawakun S Somprasit C Tanathornkeerati N . Effects of the consumption of low to medium glycemic index-based rice on the rate of insulin initiation in patients with gestational diabetes: a triple-blind, randomized, controlled trial. Clin Ther. (2023) 45:347–53. doi: 10.1016/j.clinthera.2023.02.009 36966067

[62] Nurkolis F Harbuwono DS Taslim NA Soegondo S Suastika K Sparringa RA . New insight on dietary strategies to increase insulin sensitivity and reduce diabetes prevalence: an expert perspective and recommendation. Discov Food. (2025) 5:136. doi: 10.1007/s44187-025-00422-6

[63] Nardocci M Polsky JY Moubarac JC. Consumption of ultra-processed foods is associated with obesity, diabetes and hypertension in Canadian adults. Can J Public Health Rev Can Sante Publique. (2021) 112:421–9. doi: 10.17269/s41997-020-00429-9 33174128 PMC8076355

[64] Srour B Fezeu LK Kesse-Guyot E Allès B Debras C Druesne-Pecollo N . Ultraprocessed food consumption and risk of type 2 diabetes among participants of the Nutrinet-Santé prospective cohort. JAMA Intern Med. (2020) 180:283–91. doi: 10.1001/jamainternmed.2019.5942 31841598 PMC6990737

[65] Rondinella D Raoul PC Valeriani E Venturini I Cintoni M Severino A . The detrimental impact of ultra-processed foods on the human gut microbiome and gut barrier. Nutrients. (2025) 17:859. doi: 10.3390/nu17050859 40077728 PMC11901572

[66] Fitzpatrick JA Gibson PR Taylor KM Halmos EP. The effect of dietary emulsifiers and thickeners on intestinal barrier function and its response to acute stress in healthy adult humans: a randomised controlled feeding study. Aliment Pharmacol Ther. (2024) 60:863–75. doi: 10.1111/apt.18172 39072856

[67] Raoul P Cintoni M Palombaro M Basso L Rinninella E Gasbarrini A . Food additives, a key environmental factor in the development of IBD through gut dysbiosis. Microorganisms. (2022) 10:167. doi: 10.3390/microorganisms10010167 35056616 PMC8780106

[68] Micha R Peñalvo JL Cudhea F Imamura F Rehm CD Mozaffarian D. Association between dietary factors and mortality from heart disease, stroke, and type 2 diabetes in the United States. JAMA. (2017) 317:912–24. doi: 10.1001/jama.2017.0947 28267855 PMC5852674

[69] Mozaffarian D. Dietary and policy priorities for cardiovascular disease, diabetes, and obesity: a comprehensive review. Circulation. (2016) 133:187–225. doi: 10.1161/CIRCULATIONAHA.115.018585 26746178 PMC4814348

[70] Juul F Vaidean G Lin Y Deierlein AL Parekh N. Ultra-processed foods and incident cardiovascular disease in the Framingham offspring study. J Am Coll Cardiol. (2021) 77:1520–31. doi: 10.1016/j.jacc.2021.01.047 33766258

[71] Srour B Fezeu LK Kesse-Guyot E Allès B Méjean C Andrianasolo RM . Ultra-processed food intake and risk of cardiovascular disease: prospective cohort study (NutriNet-Santé). BMJ. (2019) 365:l1451. doi: 10.1136/bmj.l1451 31142457 PMC6538975

[72] Micha R Shulkin ML Peñalvo JL Khatibzadeh S Singh GM Rao M . Etiologic effects and optimal intakes of foods and nutrients for risk of cardiovascular diseases and diabetes: Systematic reviews and meta-analyses from the Nutrition and chronic diseases expert group (NutriCoDE). PLoS ONE. (2017) 12:e0175149. doi: 10.1371/journal.pone.0175149 28448503 PMC5407851

[73] Arnett DK Blumenthal RS Albert MA Buroker AB Goldberger ZD Hahn EJ . 2019 ACC/AHA guideline on the primary prevention of cardiovascular disease: a report of the American college of cardiology/american heart association task force on clinical practice guidelines. J Am Coll Cardiol. (2019) 74:e177–232. 10.1016/j.jacc.2019.03.010 PMC7685565 30894318

[74] He FJ Tan M Ma Y MacGregor GA. Salt reduction to prevent hypertension and cardiovascular disease: JACC state-of-the-art review. J Am Coll Cardiol. (2020) 75:632–47. doi: 10.1016/j.jacc.2019.11.055 32057379

[75] Pilic L Pedlar CR Mavrommatis Y. Salt-sensitive hypertension: mechanisms and effects of dietary and other lifestyle factors. Nutr Rev. (2016) 74:645–58. doi: 10.1093/nutrit/nuw028 27566757

[76] Jalali M Bahadoran Z Mirmiran P Khalili D Symonds ME Azizi F . Higher ultra-processed food intake is associated with an increased incidence risk of cardiovascular disease: the Tehran lipid and glucose study. Nutr Metab. (2024) 21:14. doi: 10.1186/s12986-024-00788-x 38504359 PMC10949749

[77] Lane MM Gamage E Du S Ashtree DN McGuinness AJ Gauci S . Ultra-processed food exposure and adverse health outcomes: umbrella review of epidemiological meta-analyses. BMJ. (2024) 384:e077310. doi: 10.1136/bmj-2023-077310 38418082 PMC10899807

[78] Ndumele CE Rangaswami J Chow SL Neeland IJ Tuttle KR Khan SS . Cardiovascular-kidney-metabolic health: a presidential advisory from the American heart association. Circulation. (2023) 148:1606–35. doi: 10.1161/CIR.0000000000001184 37807924

[79] Bansal N Weiner D Sarnak M. Cardiovascular-kidney-metabolic health syndrome. J Am Soc Nephrol JASN. (2024) 35:649–52. doi: 10.1681/ASN.0000000000000323 38421033 PMC11149035

[80] Nie C Li Y Qian H Ying H Wang L. Advanced glycation end products in food and their effects on intestinal tract. Crit Rev Food Sci Nutr. (2022) 62:3103–15. doi: 10.1080/10408398.2020.1863904 33356474

[81] Feng JX Hou FF Liang M Wang GB Zhang X Li HY . Restricted intake of dietary advanced glycation end products retards renal progression in the remnant kidney model. Kidney Int. (2007) 71:901–11. doi: 10.1038/sj.ki.5002162 17342181

[82] Fotheringham AK Gallo LA Borg DJ Forbes JM. Advanced glycation end products (AGEs) and chronic kidney disease: does the modern diet age the Kidney? Nutrients. (2022) 14:2675. doi: 10.3390/nu14132675 35807857 PMC9268915

[83] Abd-Elhakim YM Behairy A Hashem MMM Abo-El-Sooud K El-Metwally AE Hassan BA . Toll-like receptors and nuclear factor kappa B signaling pathway involvement in hepatorenal oxidative damage induced by some food preservatives in rats. Sci Rep. (2023) 13:5938. doi: 10.1038/s41598-023-32887-9 37045926 PMC10097866

[84] Yang T Richards EM Pepine CJ Raizada MK. The gut microbiota and the brain-gut-kidney axis in hypertension and chronic kidney disease. Nat Rev Nephrol. (2018) 14:442–56. doi: 10.1038/s41581-018-0018-2 29760448 PMC6385605

[85] Evenepoel P Poesen R Meijers B. The gut-kidney axis. Pediatr Nephrol. (2017) 32:2005–14. doi: 10.1007/s00467-016-3527-x 27848096

[86] Du S Kim H Crews DC White K Rebholz CM. Association Between ultraprocessed food consumption and risk of incident CKD: a prospective cohort study. Am J Kidney Dis. (2022) 80:589-598.e1. doi: 10.1053/j.ajkd.2022.03.016 35679994 PMC9613500

[87] He X Zhang X Si C Feng Y Zhu Q Li S . Ultra-processed food consumption and chronic kidney disease risk: a systematic review and dose-response meta-analysis. Front Nutr. (2024) 11:1359229. doi: 10.3389/fnut.2024.1359229 38606016 PMC11007045

[88] Investigators CS. Ultraprocessed foods and kidney disease progression, mortality, and cardiovascular disease risk in the CRIC study. Am J Kidney Dis. (2023) 82:202–12. doi: 10.1053/j.ajkd.2023.01.452 37028638 PMC10524102

[89] Vieira de Oliveira Salerno PR Gonzalez A Hum A Baur A Carpenter C Bassiony M . Neighborhood walkability and cardio-kidney-metabolic syndrome: a narrative review. J Vasc Dis. (2025) 9. doi: 10.3390/jvd4010009

[90] Frank LD Andresen MA Schmid TL. Obesity relationships with community design, physical activity, and time spent in cars. Am J Prev Med. (2004) 27:87–96. doi: 10.1016/j.amepre.2004.04.011 15261894

[91] Sallis JF Glanz K. Physical activity and food environments: solutions to the obesity epidemic. Milbank Q. (2009) 87:123–54. doi: 10.1111/j.1468-0009.2009.00550.x 19298418 PMC2879180

[92] Sharkey JR Horel S Han D Huber JC. Association between neighborhood need and spatial access to food stores and fast food restaurants in neighborhoods of Colonias. Int J Health Geogr. (2009) 8:9. doi: 10.1186/1476-072X-8-9 19220879 PMC2653484

[93] Creatore MI Glazier RH Moineddin R Fazli GS Johns A Gozdyra P . Association of Neighborhood Walkability With Change in Overweight, Obesity, and Diabetes. JAMA. (2016) 315:2211–20. doi: 10.1001/jama.2016.5898 27218630

[94] Morland K Wing S Diez Roux A Poole C. Neighborhood characteristics associated with the location of food stores and food service places. Am J Prev Med. (2002) 22:23–9. doi: 10.1016/S0749-3797(01)00403-2 11777675

[95] Cummins S Petticrew M Sparks L Findlay A. Large scale food retail interventions and diet. BMJ. (2005) 330:683–4. doi: 10.1136/bmj.330.7493.683 15790619 PMC555618

[96] Rajagopalan S Landrigan PJ. Pollution and the Heart. N Engl J Med. (2021) 385:1881–92. doi: 10.1056/NEJMra2030281 34758254

[97] Al-Kindi SG Brook RD Biswal S Rajagopalan S. Environmental determinants of cardiovascular disease: lessons learned from air pollution. Nat Rev Cardiol. (2020) 17:656–72. doi: 10.1038/s41569-020-0371-2 32382149 PMC7492399

[98] Hajat A Hsia C O'Neill MS. Socioeconomic Disparities and Air Pollution Exposure: a Global Review. Curr Environ Health Rep. (2015) 2:440–50. doi: 10.1007/s40572-015-0069-5 26381684 PMC4626327

[99] Macias T. Environmental risk perception among race and ethnic groups in the United States. Ethnicities. (2016) 16:111–29. doi: 10.1177/1468796815575382

[100] Rocha-Velasco OA Morales-Suárez-Varela M Llopis-González A. Dietary Flavonoids: Mitigating Air Pollution's Cardiovascular Risks. Nutrients. (2024) 16:2647. doi: 10.3390/nu16162647 39203784 PMC11356943

[101] Tong H. Dietary and pharmacological intervention to mitigate the cardiopulmonary effects of air pollution toxicity. Biochim Biophys Acta. (2016) 1860:2891–8. doi: 10.1016/j.bbagen.2016.05.014 27189803

[102] Intergovernmental Panel on Climate Change (IPCC). Climate Change 2022 – Impacts, Adaptation and Vulnerability: Working Group II Contribution to the Sixth Assessment Report of the Intergovernmental Panel on Climate Change. Cambridge: Cambridge University Press; 2023. Available online at: https://www.cambridge.org/core/books/climate-change-2022-impacts-adaptation-and-vulnerability/161F238F406D530891AAAE1FC76651BD (accessed February 5, 2026). doi: 10.1017/9781009325844

[103] Fanzo J Carducci B Louis-Jean J Herrero M Karl K Rosenzweig C. Climate change, extreme weather events, food security, and nutrition: evolving relationships and critical challenges. Annu Rev Nutr. (2025) 45:335–60 doi: 10.1146/annurev-nutr-111324-111252 40239306

[104] Arsad FS Hod R Ahmad N Ismail R Mohamed N Baharom M . The impact of heatwaves on mortality and morbidity and the associated vulnerability factors: a systematic review. Int J Environ Res Public Health. (2022) 19:16356. doi: 10.3390/ijerph192316356 36498428 PMC9738283

[105] Bouchama A Knochel JP. Heat Stroke. N Engl J Med. (2002) 346:1978–88. doi: 10.1056/NEJMra011089 12075060

[106] Vieira de. Oliveira Salerno PR Lewis PGT Chen Z Dazard J Ganatra S Ahmed EN . Climate vulnerability and cardiovascular-kidney-metabolic disease in the United States. J Am Heart Assoc. (2025) 14:e038251. doi: 10.1161/JAHA.124.038251 40417800 PMC12229176

[107] Kenny GP Yardley J Brown C Sigal RJ Jay O. Heat stress in older individuals and patients with common chronic diseases. CMAJ Can Med Assoc J. (2010) 182:1053–60. doi: 10.1503/cmaj.081050 19703915 PMC2900329

[108] Reid CE O'Neill MS Gronlund CJ Brines SJ Brown DG Diez-Roux AV . Mapping community determinants of heat vulnerability. Environ Health Perspect. (2009) 117:1730–6. doi: 10.1289/ehp.0900683 20049125 PMC2801183

[109] Sun Y Zhu S Wang D Duan J Lu H Yin H . Global supply chains amplify economic costs of future extreme heat risk. Nature. (2024) 627:797–804. doi: 10.1038/s41586-024-07147-z 38480894 PMC10972753

[110] Kroeger C. Heat is associated with short-term increases in household food insecurity in 150 countries and this is mediated by income. Nat Hum Behav. (2023) 7:1777–86. doi: 10.1038/s41562-023-01684-9 37604991 PMC10593604

[111] He C Zhu Y Bachwenkizi J Chen R Kan H Fawzi WW. Assessment of heatwave impacts on child feeding practices across 36 low-income and middle-income countries: a cross-sectional analysis. Lancet Planet Health. (2025) 9:101281. doi: 10.1016/j.lanplh.2025.06.005 40712629 PMC12447361

[112] Venn D Strazdins L. Your money or your time? How both types of scarcity matter to physical activity and healthy eating. Soc Sci Med. (2017) 172:98–106. doi: 10.1016/j.socscimed.2016.10.023 27839899

[113] Walker RE Keane CR Burke JG. Disparities and access to healthy food in the United States: a review of food deserts literature. Health Place. (2010) 16:876–84. doi: 10.1016/j.healthplace.2010.04.013 20462784

[114] Darmon N Drewnowski A. Does social class predict diet quality? Am J Clin Nutr. (2008) 87:5. doi: 10.1093/ajcn/87.5.1107 18469226

[115] Drewnowski A Specter SE. Poverty and obesity: the role of energy density and energy costs. Am J Clin Nutr. (2004) 79:6–16. doi: 10.1093/ajcn/79.1.6 14684391

[116] Hossfeld LH Rico Mendez G. Looking For Food: Food Access, Food Insecurity, and the Food Environment in Rural Mississippi. Fam Community Health. (2018) 41:S7. doi: 10.1097/FCH.0000000000000182 29461311

[117] Swope CB Hernández D Cushing LJ. The relationship of historical redlining with present-day neighborhood environmental and health outcomes: a scoping review and conceptual model. J Urban Health Bull N Y Acad Med. (2022) 99:959–83. doi: 10.1007/s11524-022-00665-z 35915192 PMC9342590

[118] Motairek I Lee EK Janus S Farkouh M Freedman D Wright J . Historical neighborhood redlining and contemporary cardiometabolic risk. J Am Coll Cardiol. (2022) 80:171–5. doi: 10.1016/j.jacc.2022.05.010 35798451 PMC10411483

[119] Steptoe A Kivimäki M. Stress and cardiovascular disease. Nat Rev Cardiol. (2012) 9:360–70. doi: 10.1038/nrcardio.2012.45 22473079

[120] de Oliveira Salerno PRV Estrada-Mendizabal RJ Lozada C Ganatra S Bassiony M Aboukhatwa O . Microplastics and cardiovascular disease: should clinicians be paying attention? Curr Cardiol Rep. (2025) 27:159. doi: 10.1007/s11886-025-02320-w 41307802

[121] Cox KD Covernton GA Davies HL Dower JF Juanes F Dudas SE. Human consumption of microplastics. Environ Sci Technol. (2019) 53:7068–74. doi: 10.1021/acs.est.9b01517 31184127

[122] Toussaint B Raffael B Angers-Loustau A Gilliland D Kestens V Petrillo M . Review of micro- and nanoplastic contamination in the food chain. Food Addit Contam Part A. (2019) 36:639–73. doi: 10.1080/19440049.2019.1583381 30985273

[123] Winiarska E Jutel M Zemelka-Wiacek M. The potential impact of nano- and microplastics on human health: Understanding human health risks. Environ Res. (2024) 251:118535. doi: 10.1016/j.envres.2024.118535 38460665

[124] Afshin A Sur PJ Fay KA . Health effects of dietary risks in 195 countries, 1990–2017: a systematic analysis for the global burden of disease study 2017. Lancet. (2019) 393:1958–72. doi: 10.1016/S0140-6736(19)30041-8 30954305 PMC6899507

[125] Dong C Huang Z Wild SH . Cardiovascular disease burden attributable to dietary risk factors from 1990 to 2019: a systematic analysis of the Global Burden of Disease Study 2019. Nutr Metab Cardiovasc Dis. (2022) 32:897–908. doi: 10.1016/j.numecd.2021.11.012 35067445

[126] Pineda E Stockton J Scholes S . Food environment and obesity: a systematic review and meta-analysis. BMJ Nutr Prev Health. (2024) 7:e000663. doi: 10.1136/bmjnph-2023-000663 38966119 PMC11221287

[127] FAO IFAD UNICEF WFP WHO. The State of Food Security and Nutrition in the World 2024. Rome: FAO; 2024.

[128] Boone-Heinonen J Gordon-Larsen P Guilkey DK Jacobs DR Jr Popkin BM. Residential self-selection bias in the estimation of built environment effects on physical activity. Int J Behav Nutr Phys Act. (2010) 7:70. doi: 10.1186/1479-5868-7-70 20920341 PMC2959083

[129] Kwan M-P. The uncertain geographic context problem in environmental health research. Ann Assoc Am Geogr. (2012) 102:958–68. doi: 10.1080/00045608.2012.687349

[130] Chen X Clark J. Measuring space–time access to food retailers: a case temporal access disparity in franklin county, ohio. The Professional Geographer. (2016) 68:175–88. doi: 10.1080/00330124.2015.1032876

[131] Siddiqui NZ. . GPS-defined food environment exposures and diet-related and cardiometabolic outcomes: a systematic review. Nutrients. (2024) 16:411. doi: 10.1186/s12942-024-00362-x 38321477 PMC10848400

[132] Swinburn B Sacks G Vandevijvere S . INFORMAS: overview and key principles. Obes Rev. (2013) 14:1–12. doi: 10.1111/obr.12087 24074206

[133] Colchero MA Popkin BM Rivera JA Ng SW. Beverage purchases from stores in Mexico under the excise tax on sugar-sweetened beverages. BMJ. (2016) 352:h6704. doi: 10.1136/bmj.h6704 26738745 PMC4986313

[134] Taillie LS Reyes M Colchero MA Popkin B Corvalán C. Evaluation of Chile's law of food labeling and advertising. PLoS Med. (2020) 17:e1003015. doi: 10.1371/journal.pmed.1003015 32045424 PMC7012389

[135] Olsho LEW Klerman JA Wilde PE Bartlett S. Financial incentives increase fruit and vegetable intake among SNAP participants. Am J Clin Nutr. (2016) 104:423–35. doi: 10.3945/ajcn.115.129320 27334234

